# Goal-directed fluid management based on pulse pressure variation monitoring during high-risk surgery: a pilot randomized controlled trial

**DOI:** 10.1186/cc6117

**Published:** 2007-09-07

**Authors:** Marcel R Lopes, Marcos A Oliveira, Vanessa Oliveira S Pereira, Ivaneide Paula B Lemos, Jose Otavio C Auler, Frédéric Michard

**Affiliations:** 1 Department of Anesthesia and Critical Care, Santa Casa de Misericórdia de Passos, 164 rua Santa Casa, 37900-020, Passos, MG, Brazil; 2 Department of Anesthesia and Critical Care, INCOR-University of São Paulo, 44 Dr. Enéas de Carvalho Aguiar Avenida, 05403-000, São Paulo, SP, Brazil; 3 Department of Anesthesia and Critical Care, Béclère Hospital – University Paris XI, 157 rue de la Porte de Trivaux, 92141, Clamart, France

## Abstract

**Introduction:**

Several studies have shown that maximizing stroke volume (or increasing it until a plateau is reached) by volume loading during high-risk surgery may improve post-operative outcome. This goal could be achieved simply by minimizing the variation in arterial pulse pressure (ΔPP) induced by mechanical ventilation. We tested this hypothesis in a prospective, randomized, single-centre study. The primary endpoint was the length of postoperative stay in hospital.

**Methods:**

Thirty-three patients undergoing high-risk surgery were randomized either to a control group (group C, *n *= 16) or to an intervention group (group I, *n *= 17). In group I, ΔPP was continuously monitored during surgery by a multiparameter bedside monitor and minimized to 10% or less by volume loading.

**Results:**

Both groups were comparable in terms of demographic data, American Society of Anesthesiology score, type, and duration of surgery. During surgery, group I received more fluid than group C (4,618 ± 1,557 versus 1,694 ± 705 ml (mean ± SD), *P *< 0.0001), and ΔPP decreased from 22 ± 75 to 9 ± 1% (*P *< 0.05) in group I. The median duration of postoperative stay in hospital (7 versus 17 days, *P *< 0.01) was lower in group I than in group C. The number of postoperative complications per patient (1.4 ± 2.1 versus 3.9 ± 2.8, *P *< 0.05), as well as the median duration of mechanical ventilation (1 versus 5 days, *P *< 0.05) and stay in the intensive care unit (3 versus 9 days, *P *< 0.01) was also lower in group I.

**Conclusion:**

Monitoring and minimizing ΔPP by volume loading during high-risk surgery improves postoperative outcome and decreases the length of stay in hospital.

**Trial registration:**

NCT00479011

## Introduction

Several reports [1-4] have shown that monitoring and maximizing stroke volume by volume loading during high-risk surgery decreases the incidence of postoperative complications and the length of stay in the intensive care unit (ICU) and in the hospital. Unfortunately, this strategy has so far required the measurement of stroke volume by a cardiac output monitor as well as a specific training period for the operators [5].

By increasing pleural pressure, mechanical inspiration induces cyclic variations in cardiac preload that may be turned into cyclic changes in left ventricular stroke volume and arterial pulse pressure (the difference between systolic and diastolic pressure) [6]. The variation in arterial pulse pressure (ΔPP) induced by mechanical ventilation is known to be a very accurate predictor of fluid responsiveness; that is, of the position on the preload/stroke volume relationship (the Frank-Starling curve) [7-11]. In brief, in patients operating on the flat portion of the Frank-Starling curve (and hence insensitive to cyclic changes in preload induced by mechanical ventilation), ΔPP is low, and volume loading does not result in a significant increase in stroke volume [6]. Conversely, in patients operating on the steep portion of the preload/stroke volume relationship (and hence sensitive to cyclic changes in preload induced by mechanical ventilation), ΔPP is high, and volume loading leads to a significant increase in stroke volume [6]. By increasing cardiac preload, volume loading induces a rightward shift on the preload/stroke volume relationship and hence a decrease in ΔPP. Patients who have reached the plateau of the Frank-Starling relationship can be identified as patients in whom ΔPP is low [6,12]. The clinical and intraoperative goal of 'maximizing stroke volume by volume loading' can therefore be achieved simply by minimizing ΔPP [12].

We performed the present study to investigate whether monitoring and minimizing ΔPP by volume loading during high-risk surgery may improve postoperative outcome.

## Materials and methods

### Patients

After approval by the ethical committee of Santa Casa de Misericórdia de Passos (Passos, MG, Brazil) and written informed consent, 33 patients undergoing high-risk surgery were enrolled between 22 September 2005 and 23 January 2006 and randomized to either a control group (group C) or an intervention group (group I). Patients were selected according to a preoperative decision (by the surgeon and the intensivist) that postoperative care would be undertaken in the ICU because of co-morbidities or/and the surgical procedure. Patients less than 18 years old, with cardiac arrhythmias, with a body mass index of more than 40, or undergoing surgery with an open thorax, neurosurgery or emergency surgery, were excluded.

### Intraoperative monitoring

**Figure 1:**
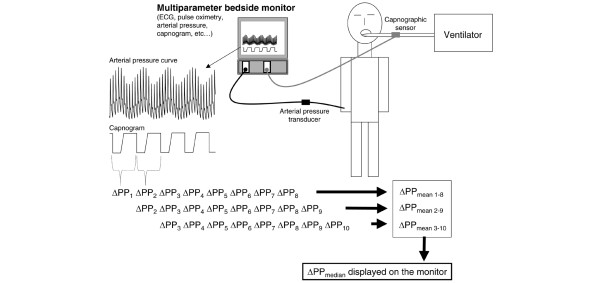
Automatic calculation of variation in arterial pulse pressure (ΔPP) from the recordings of arterial pressure and capnographic signals on a regular bedside monitor.

Heart rate, arterial pressure (radial arterial line, 20 gauge), pulse oximetry, and capnography (Capnostat Mainstream CO_2 _sensor, Respironics Inc., Murrysville, PA, USA) were monitored in all patients during the surgical procedure with the use of a multiparameter bedside monitor (DX 2020; Dixtal, São Paulo, SP, Brazil). In patients in group I, the arterial pressure curve was recorded via a specific module (IBPplus; Dixtal), allowing the automatic calculation of ΔPP by the monitor as follows (Figure 1). Each respiratory cycle is identified from the capnogram, systolic and diastolic arterial pressures are measured on a beat-to-beat basis, and pulse pressure is calculated as the difference between systolic and diastolic pressure. Maximum and minimum values for pulse pressure (PP_max _and PP_min_, respectively) are determined over each respiratory cycle, and ΔPP is calculated as a percentage as described originally [13]:

ΔPP = 100 × (PP_max _- PP_min_)/[(PP_max _+ PP_min_)/2]

The mean value of ΔPP is automatically calculated over three consecutive floating periods of eight respiratory cycles, and the median value of this triple determination is displayed on the bedside monitor and updated after each new respiratory cycle (Figure 1).

### Protocol

Randomization was performed preoperatively by using sealed envelopes. During the surgical procedure, patients were managed in accordance with our institution's standard of care. Group C received fluid intraoperatively at the discretion of the anesthetist, whereas group I received additional hydroxyethylstarch 6% (HES) boluses to minimize and maintain ΔPP ≤ 10%. This ΔPP cutoff value was chosen according to previous reports showing that when ΔPP ≤ 10%, an increase in stroke volume of 10% or more as a result of volume loading is very unlikely [7-11,13]. During the postoperative period, both groups were managed by intensivists (in the ICU), and clinicians (in the wards) not involved in the intraoperative management or in data collection. These individuals were not informed of patient allocation.

### Data collection

**Figure 2:**
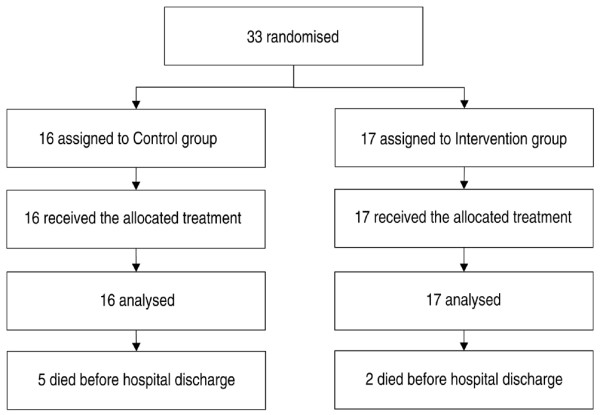
Trial profile.

Over the study period all data were collected prospectively and patients were followed up until hospital discharge. Preoperative and intraoperative data collection was undertaken by one of the investigators (VOSP), whereas postoperative data collection was undertaken by another (IPBL), who was not aware of the allocation group. Figure 2 shows the trial profile. Before surgery, the sex, age, weight, height, history of renal failure requiring dialysis or not, cirrhosis, chronic obstructive pulmonary disease, hypertension, peripheral vascular disease, coronary artery disease, other cardiac disease, diabetes mellitus, and cerebrovascular disease were recorded. The body mass index was calculated according to the standard formula (BMI = weight/height^2^). Serum creatinine concentration, prothrombin time, hemoglobin concentration, and platelet concentration were obtained from routine preoperative biological tests. During the surgical procedure, tidal volume, ventilatory frequency, infused volume of crystalloid solutions, HES, and blood products were recorded. Heart rate, mean arterial pressure, percutaneous arterial oxygen saturation, and hemoglobin concentration were collected both at the beginning and at the end of the surgical procedure. The duration of surgery was also recorded. After the surgical procedure, the following parameters were collected both at admission to the ICU and 24 hours later: mean arterial pressure, heart rate, percutaneous arterial oxygen saturation. During the 24 hours after admission to the ICU, venous lactate concentrations were measured every 6 hours and the mean lactate value was calculated over the first 24-hour period in the ICU. The need for continuous vasoactive (dopamine or/and norepinephrine (noradrenaline)) support was also recorded.

Postoperative ICU infections (pneumonia, abdominal, urinary tract, line-related sepsis and wound infections), respiratory complications (pulmonary embolism, acute lung injury, and respiratory support for more than 24 hours exclusive of acute lung injury), cardiovascular complications (arrhythmia, hypotension, acute pulmonary edema, acute myocardial infarction, stroke, and cardiac arrest exclusive of fatal outcome), abdominal complications (*Clostridium difficile *diarrhea, acute bowel obstruction, upper gastrointestinal bleed, and anastomotic leak), hematologic complications (platelet count less than 100,000/μl or prothrombin time less than 50%), and renal complications (urine output less than 500 ml/day or serum creatinine more than 170 μmol/l or dialysis for acute renal failure) were collected in accordance with criteria used previously by other investigators [3,14,15].

### Statistical analysis

Data were analysed by comparing patients in group C with those in group I on an intention-to-treat basis. The primary outcome measure was the duration of postoperative stay in hospital. On the basis of our own hospital registry, the mean duration of postoperative stay in hospital in group C was a priori estimated at 16 ± 8 days (mean ± SD). In accordance with previous publications [1,2], we postulated that the mean duration of postoperative stay in hospital in group I could be 35% lower. A sample size of 33 patients in each group was calculated for a 0.05 difference (two-sided) with a power of 80% [16]. An intermediate analysis after the enrolment of the first 33 patients was planned, to readjust the population sample size if necessary. Secondary outcome measures were the number of postoperative complications per patient, as well as the duration of mechanical ventilation and stay in the ICU.

Results are expressed as mean ± SD, or as median [interquartile ranges] for the duration of mechanical ventilation, stay in the ICU, and stay in hospital. Comparisons between groups C and I were performed with a non-parametric Mann-Whitney *U *test (quantitative data) or a χ^2 ^test (qualitative data). In group I, the effect of HES administration on ΔPP during surgery was assessed with a non-parametric Wilcoxon rank-sum test. Linear correlations were tested by using the Spearman rank method. A *P *value less than 0.05 was considered statistically significant.

## Results

Over the 4-month (22 September 2005 to 23 January 2006) enrolment study period, 237 patients were admitted to our medico-surgical ICU, 57 of these after a surgical procedure. Among these 57 postoperative patients, 33 patients fulfilled the inclusion criteria and agreed to participate in the study. Sixteen patients were randomly assigned to group C and 17 to group I (Figure 2). Thestudy was stopped after the intermediate analysis (33 patients enrolled) because we observed a significant decrease in the length of stay in hospital (primary endpoint) in group I.

### Before surgery

Before surgery, the groups were comparable in terms of sex ratio, age, weight, height, body mass index, American Society of Anesthesiology (ASA) score, type of surgery, and preoperative biological tests (Table 1). They were also comparable in terms of co-morbidities, except in regard to peripheral vascular disease, where the observed incidence was significantly higher (*P *= 0.04) in group I.

**Table 1:** Patients' characteristics before surgery

Characteristic	Group	
	
	C (*n *= 16)	I (*n *= 17)
Sex, M/F	12/4	11/6
Age (years)	62 ± 10	63 ± 16
Weight (kg)	68 ± 16	66 ± 16
Height (cm)	170 ± 8	164 ± 9
Body mass index (kg/m^2^)	23 ± 4	24 ± 5
ASA II score	3	3
ASA III score	9	8
ASA IV score	4	6
Chronic disease		
Renal failure requiring dialysis	1	0
Renal failure without dialysis^a^	5	6
Cirrhosis	0	1
Chronic obstructive pulmonary disease	6	8
Hypertension	13	13
Peripheral vascular disease	3	9^b^
Coronary artery disease	1	3
Other cardiopathy	5	8
Diabetes mellitus	5	7
Cerebrovascular disease	1	3
Preoperative biological tests		
Serum creatinine (μmol/l)	124 ± 90	132 ± 55
Prothrombin time (percentage)	87 ± 13	80 ± 19
Hemoglobin (g/dl)	11.3 ± 2.0	11.9 ± 2.5
Platelets (/μl)	305,000 ± 108,000	301,000 ± 110,000

ASA, American Society of Anesthesiology physical status; C, control; I, intervention. ^a^Serum creatinine more than 130 μmol/l; ^b^*P *< 0.05, control group versus intervention group.

### During surgery

The duration of the surgical procedure, as well as respiratory settings (tidal volume and ventilatory frequency) were comparable in both groups (Table 2). During the surgical procedure, the amount of HES and the total amount of fluid (including crystalloid, HES, and blood products) was significantly greater in group I than in group C (Table 2). None of the patients received continuous vasoactive support during surgery. In group I (ΔPP was not measured in group C), ΔPP decreased significantly from 22 ± 7% to 9 ± 1% (mean ± SD; *P *< 0.0001) over the time frame of the surgical procedure, and was 10% or less at the end of the surgical procedure in all except four patients (range 7 to 11).

**Table 2:** Type of surgery, physiologic status, and fluid administered during the surgical procedure

Parameter	Group	
	
	C (*n *= 16)	I (*n *= 17)
Type of surgery		
Upper gastrointestinal	4	4
Hepato-biliary	2	3
Lower gastrointestinal	8	10
Urology	1	0
Other	1	0
Respiratory settings		
Tidal volume (ml/kg)	9.1 ± 0.5	8.6 ± 0.6
Ventilatory frequency (/min)	13 ± 1	13 ± 1
Physiologic status at start of surgery		
Heart rate (/min)	66 ± 9	77 ± 17
Mean arterial pressure (mmHg)	96 ± 16	90 ± 18
SpO_2_(percentage)	97 ± 3	97 ± 3
ΔPP (percentage)		22 ± 7
Hemoglobin (g/dl)	11.3 ± 2.0	11.9 ± 2.5
Physiologic status at end of surgery		
Heart rate (/min)	86 ± 19	80 ± 17
Mean arterial pressure (mmHg)	68 ± 20	78 ± 14
SpO_2 _(percentage)	97 ± 3	97 ± 3
ΔPP (percentage)		9 ± 1^a^
Hemoglobin (g/dl)	9.8 ± 1.4	9.6 ± 1.6
Fluid administered		
Volume of crystalloid infused (ml)	1,563 ± 602	2,176 ± 1,060
Volume of colloid infused (ml)	0	2,247 ± 697^b^
Volume of red blood cells infused (ml)	131 ± 268	159 ± 320
Number of patients who received red blood cells	4	5
Volume of FFP infused (ml)	0	35 ± 106
Number of patients who received FFP	0	2
Total volume infused (ml)	1,694 ± 705	4,618 ± 1,557^b^
Total volume infused (ml/kg per hour)	7 ± 2	21 ± 8^b^
Duration of surgery (hours)	3.7 ± 1.4	3.9 ± 2.0

SpO_2_, percutaneous arterial oxygen saturation; ΔPP, variation in arterial pulse pressure; FFP, fresh frozen plasma; C, control; I, intervention. ^a^*P *< 0.05, end of surgery versus start of surgery; ^b^*P *< 0.0001, control group versus intervention group.

### After surgery

**Figure 3:**
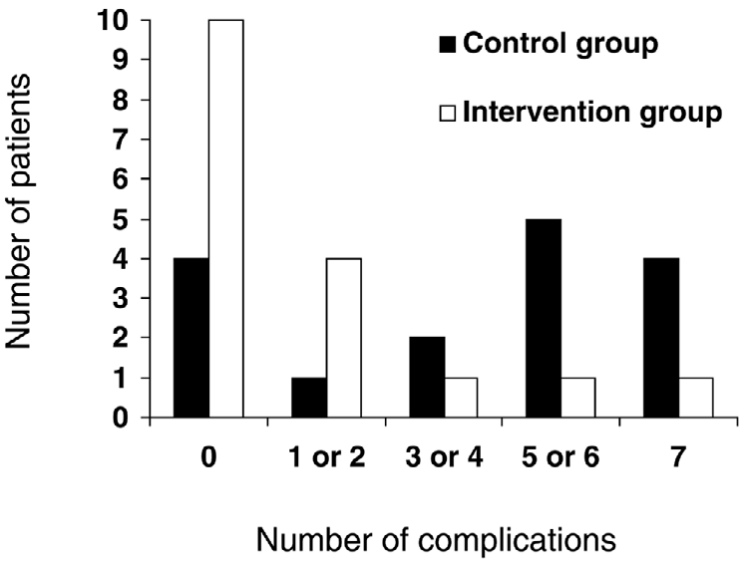
Numbers of patients with postoperative complications in the control and intervention groups.

**Figure 4:**
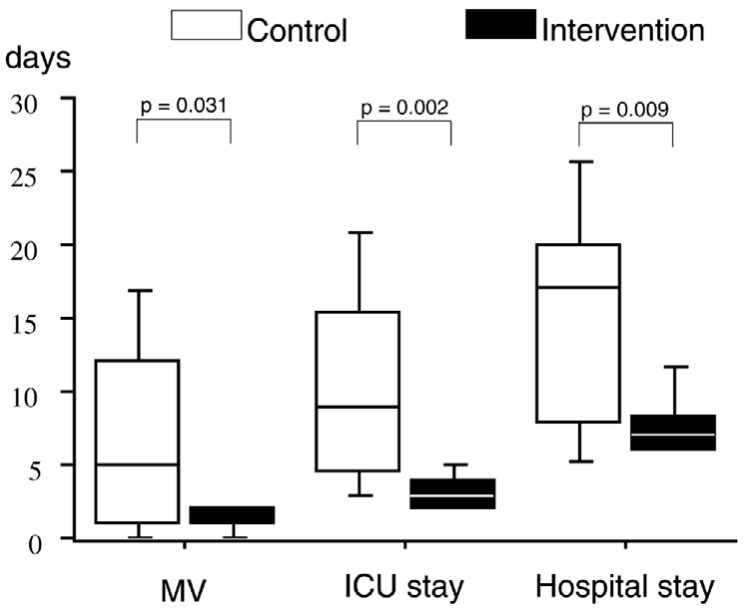
Box-and-whiskers representation of the duration (days) of mechanical ventilation (MV), stay in the intensive care unit (ICU), and stay in hospital in the control and intervention groups. The line inside a box denotes the median, the limits of the box denote the 75th centile of the data, and the whiskers represent the 90th centile of the data.

On admission to the ICU, the mean arterial pressure was significantly greater in group I (Table 3); 24 hours after admission to the ICU, fewer patients required vasoactive support in group I, and blood lactate was lower in this group (Table 3). Postoperative complications are listed in Table 4. The number of patients with postoperative complications is shown in Figure 3. Fewer patients developed complications in group I (7 patients (41%) versus 12 patients (75%), *P *= 0.049). The number of complications per patient was lower in group I than in group C (1.4 ± 2.1 per patient versus 3.9 ± 2.8 per patient, *P *= 0.015). The median [interquartile range] duration of mechanical ventilation (1 [1 to 2] versus 5 [1 to 12] days, *P *< 0.05), stay in the ICU (3 [2 to 4] versus 9 [4.5 to 15.5] days, *P *< 0.01), and stay in hospital (7 [6 to 8.25] versus 17 [8 to 20] days, *P *< 0.01) was significantly lower in group I than in group C (Figure 4). Over the study period (until hospital discharge), five patients died (on days 7, 11, 18, 19, and 26) in group C, whereas two patients died (on days 7 and 22) in group I (*P *= 0.171). In group C, the cause of death was septic shock and ARDS in four cases (pneumonia *n *= 1, abdominal sepsis *n *= 2, pneumonia and urosepsis *n *= 1), and acute pulmonary edema in one case. In group I, the cause of death was unexplained cardiac arrest in one case, and acute respiratory failure in one case (tracheostomy complication). Because death does influence the duration of mechanical ventilation, the duration of stay in the ICU, and the duration of stay in hospital, we also compared these parameters when considering only survivors (*n *= 26). The median [interquartile range] duration of mechanical ventilation, stay in the ICU, and stay in hospital was 1 [1 to 2] versus 2 [0.25 to 5.5] days (*P *= 0.29), 3 [2.25 to 4] versus 6 [3.25 to 11.75] days (*P *= 0.014), and 7 [6 to 8] versus 16 [7.5 to 20.25] days (*P *= 0.024) in survivors of group I (*n *= 15) and group C (*n *= 11), respectively.

**Table 3:** Hemodynamic and physiologic status on admission to ICU and 24 hours later

Status	Group	
	
	C (*n *= 16)	I (*n *= 17)
On admission to ICU		
Mean arterial pressure (mmHg)	66 ± 20	80 ± 18^a^
Heart rate (/min)	90 ± 18	85 ± 20
SpO_2 _(percentage)	96 ± 4	96 ± 2
Lactate (mmol/l)	1.5 ± 1.1	1.1 ± 0.8
At 24 h after admission to ICU		
Mean arterial pressure (mmHg)	80 ± 12	82 ± 11
Heart rate (/min)	92 ± 21	85 ± 18
SpO_2 _(%)	97 ± 3	95 ± 3
Vasoactive support (n)	8	2^a^
Lactate (mmol/l)	1.9 ± 1.1	0.7 ± 0.8^b^
Mean lactate over 24 h (mmol/l)	2.4 ± 1.1	1.2 ± 0.4^c^

ICU, intensive care unit; SpO_2_, percutaneous arterial oxygen saturation; C, control; I, intervention. ^a^*P *< 0.05, ^b^*P *< 0.01, ^c^*P *< 0.001, control group versus intervention group.

**Table 4:** Postoperative complications

Complication	Group	
	
	C (*n *= 16)	I (*n *= 17)
Infection		
Pneumonia	5	2
Abdominal	4	3
Urinary tract	1	0
Respiratory		
Pulmonary embolism	1	0
Respiratory support > 24 h (exclusive of acute lung injury)	6	5
Acute lung injury	5	1
Cardiovascular		
Arrhythmia^a^	6	3
Hypotension^a^	11	3
Acute pulmonary edema	2	0
Cardiac arrest (exclusive of fatal outcome)	1	0
Abdominal		
Acute bowel obstruction	1	0
Upper gastrointestinal bleed	2	1
Anastomotic leak	1	0
Coagulopathy		
Platelet count <100,000/μl^b ^or prothrombin time < 50%^c^	6	4
Renal		
Urine output < 500 ml/day or serum creatinine > 170 μmol/l^d ^or dialysis for acute renal failure	11	1
Total number of complications	63	23
Number (percentage) of patients with complications	12 (75)	7 (41)

C, control; I, intervention. ^a^Requiring pharmacologic treatment; ^b^if at least 150,000/μl preoperatively; ^c^if at least 70% preoperatively; ^d^if 130 μmol/l or less preoperatively.

## Discussion

Our study shows that monitoring and minimizing ΔPP by fluid loading during high-risk surgery decreases the incidence of postoperative complications and also the duration of mechanical ventilation, stay in the ICU, and stay in hospital.

Hypovolemia can pass undetected before, during, and after major surgery. Aside from the inevitable losses in the intraoperative period mainly due to bleeding, most patients are still starved for a minimum of 6 hours preoperatively to reduce the risk of acid aspiration syndrome. Additionally, patients undergoing abdominal surgery frequently receive bowel preparation, another factor that may induce or worsen hypovolemia [17,18]. In our study population, all patients undergoing bowel surgery (*n *= 18) received a bowel preparation (2,000 ml of mannitol solution *per os*) administered over a period of 2–3 hours and started 16 hrs before the surgical procedure, and 2,500 ml of glucose solution intravenously over the same period. Other patients (*n *= 15) were starved for 12 hours before the surgical procedure and received 1,500 ml of glucose solution intravenously over this period. Classical cardiovascular parameters such as heart rate and arterial pressure are poor indicators of volume status, and these were in the normal range in both groups just before surgery. In contrast, in comparison with values reported previously [7-11], preoperative ΔPP values were quite high (in group I), suggesting that some of our patients were probably hypovolemic at the beginning of the surgical procedure.

Perioperative hypovolemia leading to poor organ perfusion is thought to be a major factor in determining postoperative morbidity after major surgery. Optimization of circulatory status perioperatively was a concept first promulgated by Shoemaker and colleagues [19]. They found a significant reduction in mortality and stay in hospital in high-risk surgical patients receiving fluid loading with or without dobutamine to increase cardiac output and oxygen delivery to supranormal values. Comparable results from other groups [20-22] using a similar goal-directed approach lends further support to the importance of avoiding hypovolemia and tissue oxygen debt perioperatively.

Instead of targeting a given threshold value of cardiac index or of oxygen delivery during surgery, other authors have proposed to guide intraoperative fluid administration by using individual Frank-Starling curves [1-4,12,23]. Several studies have shown that monitoring and maximizing stroke volume by fluid loading (until stroke volume reaches a plateau, actually the plateau of the Frank-Starling curve) during high-risk surgery is associated with improved postoperative outcome [1-4]. The benefit in using such a fluid strategy, guided by the continuous esophageal Doppler measurement of stroke volume, was established first in patients undergoing cardiac surgery [1] or hip surgery [2], and was extended more recently to patients undergoing major bowel or general surgery [3,4].

Intra-arterial blood pressure monitoring is common practice in most patients undergoing high-risk surgery [24]. The assessment of ΔPP is therefore a simple and cost-saving method in comparison with technologies monitoring cardiac output or oxygen delivery. Such a simple approach therefore has the potential for widespread application because it is not routinely feasible for anesthetists to monito cardiac output or oxygen delivery in many institutions, as well as in many countries.

Our study has some limitations. First, this is a single-centre trial, and local perioperative standard of care may have influenced the results. There is no specific fluid protocol for high-risk surgery in Santa Casa Misericordia hospital. Anesthetists were free to use the type and the volume of fluid they considered necessary to maintain blood pressure during the surgical procedure, and did not monitor central venous pressure. As a result, group C did not receive HES and received much less fluid than group I during the surgical procedure (the difference between groups was 2,924 ml). The debate over correct intraoperative fluid management is unresolved [23,25,26]. Indeed, facing studies showing a benefit in optimizing stroke volume and oxygen delivery by fluid loading, few studies have conversely shown a benefit in fluid restriction [27-29]. For instance, Nisanevich and colleagues [29] recently compared the postoperative outcome of two groups of patients undergoing abdominal surgery, a restrictive group (receiving 4 ml/kg of crystalloid solution per hour during the surgical procedure) and a liberal group (receiving a bolus of 10 ml/kg followed by 12 ml/kg per hour during surgery). Patients in the restrictive group received an average total volume of 1,230 ml during the surgical procedure, whereas those in the liberal group received 3,670 ml (that is, 2,440 ml more). The number of patients with complications was smaller in the restrictive group, as was the duration of postoperative stay in hospital. Although the study populations are not comparable (ASA scores were higher in our study), it is interesting to note that the total amount of fluid received intraoperatively by our control group (7 ml/kg per hour) was higher than the volume of fluid received by the restrictive group (4 ml/kg per hour) of Nisanevich's study [29].

The mortality rate was high in our control group, but we must bear in mind that it was calculated from a small patient population and that most of our patients had many co-morbidities (ASA score was 3 or more in all except six patients; that is, in 82% of our study population). Moreover, it was consistent with mortality rates of patients undergoing high-risk surgery reported previously in Brazil [21,30]. In Europe or in the USA, high-risk surgery mortality rates are usually lower [3,4,15,22], although mortality rates up to 22% [20] and 34% [19] have also been reported. In this respect, our findings strongly suggest that an intraoperative goal-directed fluid therapy based on ΔPP monitoring is useful for improving outcome at least in our institution, but caution should be exercised before extrapolating our findings to other patient populations or to other institutions in which standard perioperative fluid management may be different.

The morbidity was high in our patients, with an incidence of postoperative complications of 41% and 75% in groups I and C, respectively. The overall management of our patients may have contributed, at least in part, to this finding. However, one must point out that the incidence of postoperative complications is also directly influenced by the number of complications collected. We used a very extensive list of postoperative complications, including infectious, respiratory, cardiovascular, and abdominal complications proposed recently by Pearse and colleagues [15], as well as hematologic and renal complications proposed by Bennett-Guerrero and colleagues [14] and Gan and colleagues [3]. Finally, the incidence of postoperative complications in our study was comparable to the incidence reported by Pearse and colleagues [15] in a recent study investigating the value of postoperative optimization in patients undergoing high-risk surgery (44% in the optimization group versus 68% in the control group).

The small number of patients enrolled in this study is also a limitation. Although patients were randomized, we observed that the groups were not comparable in terms of peripheral vascular disease (the incidence was higher in group I). If this finding could not be an advantage to group I, in which a better outcome was finally reported, it indicates the risk of imbalance between the groups as a result of the small sample size. In this regard, because we did not measure ΔPP in the control group, we cannot definitely exclude the possibility that ΔPP might have been different between groups C and I at the beginning of surgery. Our results therefore merit confirmation on a larger scale, and ideally on a multicentre basis. Such a trial is currently ongoing in several hospitals in São Paulo, Brazil. In contrast, the fact that we observed significant differences between the outcomes of two small groups of patients emphasizes the potential value of using ΔPP to tailor fluid administration during high-risk surgery, and the likelihood of observing similar differences in larger populations of patients.

Finally, because ΔPP is directly influenced by the magnitude of cyclic changes in pleural pressure induced by mechanical inspiration, it cannot be recommended as a guide to fluid administration in patients who are not mechanically ventilated with regular tidal volume (for example patients undergoing surgery under regional anesthesia) or when chest compliance is abnormally increased (for example during open chest surgery) or decreased (for example in morbidly obese patients) [6]. In this regard, it must be noted that these populations were excluded from the present study, as were patients with cardiac arrhythmia, in whom ΔPP cannot be evaluated [31].

## Conclusion

Our study shows that monitoring and minimizing ΔPP by volume loading during high-risk surgery decreases the number of postoperative complications and also the duration of mechanical ventilation, stay in the ICU, and stay in hospital. Thus, ΔPP may serve as a simple tool for improving the outcome of patients undergoing high-risk surgery. Further studies are required to confirm the results of our pilot study on a larger scale, as well as in different settings.

## Key messages

• Monitoring and minimizing arterial pulse pressure variation (ΔPP) by volume loading during high-risk surgery decreases the duration of stay in hospital.

• This goal-directed strategy is also useful in decreasing the number of postoperative complications, as well as the duration of mechanical ventilation and stay in the ICU.

## Abbreviations

ASA = American Society of Anesthesiology; ΔPP = variation in arterial pulse pressure; HES = hydroxyethylstarch; ICU = intensive care unit.

## Competing interests

The named authors declare that they have no conflict of interest. Dixtal had no role in the study design, data collection, data analysis, data interpretation, or writing of the report.

## Authors' contributions

FM, MRL, and JOCA participated in the trial design. VOSP and IPBL obtained the data. MRL, FM, and MAO participated in the data analysis and interpretation of the results. FM and MRL were involved in the statistical analysis and wrote the paper. All authors read and approved the final manuscript.

## References

[B1] Mythen MG, Webb AR (1995). Perioperative plasma volume expansion reduces the incidence of gut mucosal hypoperfusion during cardiac surgery. Arch Surg.

[B2] Sinclair S, James S, Singer M (1997). Intraoperative intravascular volume optimisation and length of hospital stay after repair of proximal femoral fracture: a randomised controlled trial. BMJ.

[B3] Gan TJ, Soppitt A, Maroof M, El-Moalem H, Robertson KM, Moretti E, Dwane P, Glass PSA (2002). Goal-directed intraoperative fluid administration reduces length of hospital stay after major surgery. Anesthesiology.

[B4] Wakeling HG, McFall MR, Jenkins CS, Woods WGA, Miles WFA, Barclay GR, Fleming SC (2005). Intraoperative oesophageal Doppler guided fluid management shortens postoperative hospital stay after major bowel surgery. Br J Anaesth.

[B5] Lefrant JY, Bruelle P, Aya AG, Saissi G, Dauzat M, de La Coussaye JE, Eledjam JJ (1998). Training is required to improve the reliability of esophageal Doppler to measure cardiac output in critically ill patients. Intensive Care Med.

[B6] Michard F (2005). Changes in arterial pressure during mechanical ventilation. Anesthesiology.

[B7] Michard F, Boussat S, Chemla D, Anguel N, Mercat A, Lecarpentier Y, Richard C, Pinsky MR, Teboul JL (2000). Relation between respiratory changes in arterial pulse pressure and fluid responsiveness in septic patients with acute circulatory failure. Am J Respir Crit Care Med.

[B8] Bendjelid K, Suter PM, Romand JA (2004). The respiratory change in preejection period: a new method to predict fluid responsiveness. J Appl Physiol.

[B9] Kramer A, Zygun D, Hawes H, Easton P, Ferland A (2004). Pulse pressure variation predicts fluid responsiveness following coronary artery bypass surgery. Chest.

[B10] De Backer D, Heenen S, Piagnerelli M, Koch M, Vincent JL (2005). Pulse pressure variations to predict fluid responsiveness: influence of tidal volume. Intensive Care Med.

[B11] Solus-Biguenet H, Fleyfel M, Tavernier B, Kipnis E, Onimus J, Robin E, Lebuffe G, Decoene C, Pruvot FR, Vallet B (2006). Non-invasive prediction of fluid responsivenss during major hepatic surgery. Br J Anaesth.

[B12] Michard F, Lopes MR, Auler JOC (2007). Pulse pressure variation: beyond the fluid management of patients with shock. Crit Care.

[B13] Michard F, Chemla D, Richard C, Wysocki M, Pinsky MR, Lecarpentier Y, Teboul JL (1999). Clinical use of respiratory changes in arterial pulse pressure to monitor the hemodynamic effects of PEEP. Am J Respir Crit Care Med.

[B14] Bennett-Guerrero E, Welsby I, Dunn TJ, Young LR, Wahl TA, Diers TL, Phillips-Bute BG, Newman MF, Mythen MG (1999). The use of a postoperative morbidity survey to evaluate patients with prolonged hospitalization after routine, moderate-risk, elective surgery. Anesth Analg.

[B15] Pearse R, Dawson D, Fawcett J, Rhodes A, Grounds RM, Bennett ED (2005). Early goal-directed therapy after major surgery reduces complications and duration of hospital stay. A randomised, controlled trial. Crit Care.

[B16] Schulz KF, Grimes DA (2005). Sample size calculations in randomised trials: mandatory and mystical. Lancet.

[B17] Holte K, Nielsen KG, Madsen JL, Kehlet H (2004). Physiologic effects of bowel preparation. Dis Colon Rectum.

[B18] Junghans T, Neuss H, Strohauer M, Raue W, Haase O, Schink T, Schwenk W (2006). Hypovolemia after traditional preoperative care in patients undergoing colonic surgery is underrepresented in conventional hemodynamic monitoring. Int J Colorectal Dis.

[B19] Shoemaker WC, Appel PL, Kram HB, Waxman K, Lee TS (1988). Prospective trial of supranormal values of survivors as therapeutic goals in high-risk surgical patients. Chest.

[B20] Boyd O, Grounds M, Bennett ED (1993). A randomized clinical trial of the effect of deliberate perioperative increase of oxygen delivery on mortality in high-risk surgical patients. JAMA.

[B21] Lobo SMA, Salgado PF, Castillo VG, Borim AA, Polachini CA, Palchetti JC, Brienzi SLA, de Oliveira GG (2000). Effects of maximizing oxygen delivery on morbidity and mortality in high-risk surgical patients. Crit Care Med.

[B22] Kern JW, Shoemaker WC (2002). Meta-analysis of hemodynamic optimization in high-risk patients. Crit Care Med.

[B23] Spahn DR, Chassot PG (2006). CON: fluid restriction for cardiac patients during major noncardiac surgery should be replaced by goal-directed intravascular fluid administration. Anesth Analg.

[B24] Buhre W, Rossaint R (2003). Perioperative management and monitoring in anaesthesia. Lancet.

[B25] Joshi GP (2005). Intraoperative fluid restriction improves outcome after major elective gastrointestinal surgery. Anesth Analg.

[B26] Boldt J (2006). Fluid management of patients undergoing abdominal surgery – more questions than answers. Eur J Anaesth.

[B27] Kita T, Mammoto T, Kishi Y (2002). Fluid management and postoperative respiratory disturbances in patients with transthoracic esophagectomy for carcinoma. J Clin Anesth.

[B28] Brandstrup B, Tonnesen H, Beier-Holgersen R, Hjortso E, Ording H, Lindorff-Larsen K, Rasmussen MS, Lanng C, Wallin L, the Danish Study Group on Perioperative Fluid Therapy (2003). Effects of intravenous fluid restriction on postoperative complications: comparison of two perioperative fluid regimens. Ann Surg.

[B29] Nisanevich V, Felsenstein I, Almogy G, Weissman C, Einav S, Matot I (2005). Effect of intraoperative fluid management on outcome after intraabdominal surgery. Anesthesiology.

[B30] Lobo SM, Lobo FR, Polachini CA, Patini DS, Yamamoto AE, de Oliveira NE, Serrano P, Sanches HS, Spegiorin MA, Queiroz MM (2006). Prospective, randomized trial comparing fluids and dobutamine optimization of oxygen delivery in high-risk surgical patients. Crit Care.

[B31] Michard F (2005). Volume management using dynamic parameters: the good, the bad, and the ugly. Chest.

